# Iron in Translation: From the Beginning to the End

**DOI:** 10.3390/microorganisms9051058

**Published:** 2021-05-13

**Authors:** Antonia María Romero, María Teresa Martínez-Pastor, Sergi Puig

**Affiliations:** 1 Departamento de Biotecnología, Instituto de Agroquímica y Tecnología de Alimentos (IATA), Consejo Superior de Investigaciones Científicas (CSIC), Catedrático Agustín Escardino 7, Paterna, 46980 Valencia, Spain; am.romero@iata.csic.es; 2 Departamento de Bioquímica y Biología Molecular, Universitat de València, Doctor Moliner 50, Burjassot, 46100 Valencia, Spain

**Keywords:** translation, tRNA modification, yeast, *Saccharomyces cerevisiae*, iron deficiency

## Abstract

Iron is an essential element for all eukaryotes, since it acts as a cofactor for many enzymes involved in basic cellular functions, including translation. While the mammalian iron-regulatory protein/iron-responsive element (IRP/IRE) system arose as one of the first examples of translational regulation in higher eukaryotes, little is known about the contribution of iron itself to the different stages of eukaryotic translation. In the yeast *Saccharomyces cerevisiae*, iron deficiency provokes a global impairment of translation at the initiation step, which is mediated by the Gcn2-eIF2α pathway, while the post-transcriptional regulator Cth2 specifically represses the translation of a subgroup of iron-related transcripts. In addition, several steps of the translation process depend on iron-containing enzymes, including particular modifications of translation elongation factors and transfer RNAs (tRNAs), and translation termination by the ATP-binding cassette family member Rli1 (ABCE1 in humans) and the prolyl hydroxylase Tpa1. The influence of these modifications and their correlation with codon bias in the dynamic control of protein biosynthesis, mainly in response to stress, is emerging as an interesting focus of research. Taking *S. cerevisiae* as a model, we hereby discuss the relevance of iron in the control of global and specific translation steps.

## 1. Introduction

Iron is an indispensable micronutrient for all eukaryotes and most prokaryotes due to its participation as a redox cofactor in fundamental cellular processes, including DNA replication and repair, cellular respiration, photosynthesis, oxygen transport, lipid metabolism, and, importantly, translation, which is the focus of the present review. While ferrous iron (Fe^2+^) was highly abundant and soluble in the anoxic environments where the first organisms evolved, the rise in oxygen levels in the Earth’s atmosphere compromised the bioavailability of iron, due to its oxidation to ferric iron (Fe^3+^), which is extremely insoluble at physiological pH. Therefore, organisms have evolved sophisticated mechanisms to acquire iron and modulate its homeostasis in order to maintain many essential iron-dependent processes. Nonetheless, iron deficiency is the most common nutritional disorder that humans and plants address, with important consequences for human health and the nutritional quality of crops [1,2].

It has been known for years that mitochondria are indispensable for eukaryotic life. This essentiality was first assigned to the multiple iron-dependent metabolic processes that take place in this organelle, including respiration. However, when this question was addressed more profoundly in the eukaryotic model *Saccharomyces cerevisiae*, which can grow in the absence of mitochondrial DNA, it was discovered that the minimal essential function of mitochondria relied on the biogenesis of cellular iron-sulfur (Fe/S) proteins (reviewed in [3]). Intriguingly, the first essential target of the mitochondrial Fe/S cluster (ISC) assembly system identified was a cytosolic protein, Rli1 (ABCE1 in humans), which is involved in ribosome biogenesis and several steps of the translational process, and contains ISCs essential for its multiple functions (see below) [3,4,5,6]. More recently, essential ISCs have been also identified in all replicative DNA polymerases [7,8]. In addition to the highly conserved ABCE1/Rli1 protein, recent studies have revealed the involvement of additional ISC-containing proteins in numerous steps of translation, underscoring the importance of this element for this process and raising the question of how iron deficiency affects translation. Using the yeast *S. cerevisiae* as a model, in this review, we update the answers we actually have to these questions. Thus, recent studies have determined that, under short exposure to iron deficiency, yeast cells sustain global translation levels. However, if iron deprivation persists, a global translational arrest occurs at the initiation step, which is regulated by the TORC1 and Gcn2/eIF2α pathways, while the translation of a subset of messenger RNAs (mRNAs) encoding iron-containing proteins mainly involved in non-essential processes is inhibited through the post-translational regulator Cth2 [9,10,11,12,13]. We also focus on the different iron-containing proteins that participate, either directly or indirectly, in the translation process and its regulation, with special emphasis on modifications of factors affecting translational elongation (such as diphthamide modification of eEF2 and hypusination of eIF5A), post-transcriptional transfer RNA (tRNA) modifications (Elp3 and Dph3/Kti11 in the Elongator complex, wybutosine tRNA modification by Tyw1), and translational termination (Tpa1) (see Table 1). Protein homologs in humans or other organisms, as well as related human diseases caused by their deficiencies, will be discussed when they are deemed relevant.

## 2. Iron Deficiency Impairs Translation at the Initiation Step

**Figure 1:**
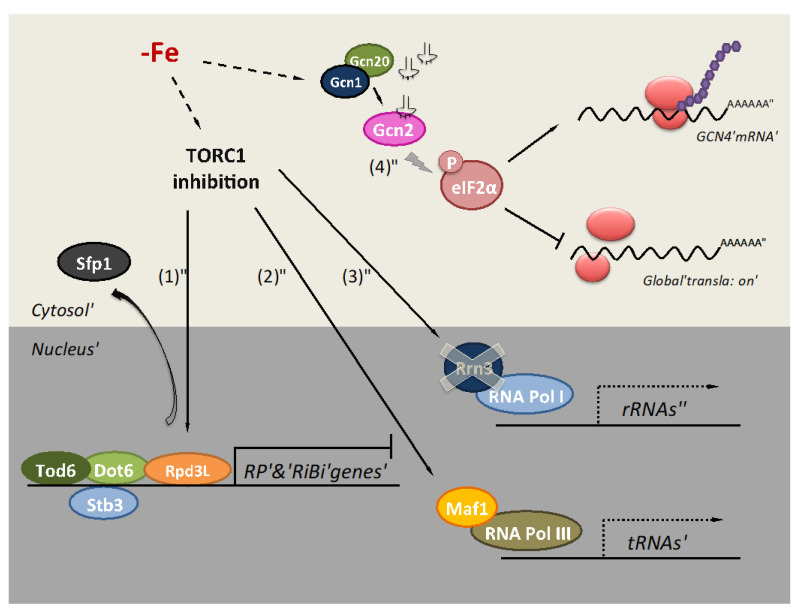
Mechanisms of regulation of protein synthesis in response to iron deficiency in the budding yeast *S. cerevisiae* at different levels. Upon iron depletion, TORC1 complex is inactivated, leading to (1) repression of RP and RiBi genes transcription by Tod6, Dot6, Stb3, and Rpd3L complex, and export of their transcriptional activator Sfp1 to the cytosol; (2) inhibition of RNA Pol III by association to its dephosphorylated Maf1 repressor; and (3) inhibition of RNA Pol I activity due to the decrease in the levels of its Rnr3 activator [12]. Moreover, (4) phosphorylation of eIF2α by Gcn2 inhibits global protein synthesis at the initiation stage in a Gcn1-Gcn20-dependent manner and promotes *GCN4* mRNA translation [36].

Protein translation is a crucial process in the modulation of gene expression, being one of the most energy-consuming cellular processes. Cells need to adapt their proteome to survive in response to adverse conditions. Thus, translation inhibition has been described in response to several stresses including osmotic and oxidative stress, heat shock, and nutritional deficiencies of glucose, amino acids, and, more recently, iron [12,28,29,30,31]. Translation is an iron-dependent process because the biogenesis and recycling of ribosomes depend on the essential and conserved Fe/S protein ABCE1/Rli1 (see below). Furthermore, multiple microbial amino acid biosynthesis pathways, including leucine, isoleucine, valine, lysine, glutamate, and methionine utilize iron-dependent enzymes. The TORC1 pathway is the main process integrating the environmental signals to regulate protein translation and other metabolic processes. When cells are exposed to unfavorable growth conditions, the TORC1 complex is inactivated, promoting an adaptation of the gene expression program [32]. Recently, inhibition of yeast TORC1 during iron deficiency has been characterized at multiple levels by genome-wide and process-specific analyses (Figure 1) [12]. In response to low iron availability, cells diminish RNA polymerase (RNA Pol) II transcription rate and decrease the abundance of many mRNAs encoding for ribosomal proteins (RP) and ribosome biogenesis (RiBi) factors. Multiple TORC1-modulated pathways regulate the transcription of RP and RiBi genes (Figure 1) [12]. Yeast TORC1 inactivation provokes a decrease in the phosphorylation of the protein kinase Sch9 and, subsequently, the activation of the repressors Stb3 and Dot6/Tod6, and Rpd3L histone deacetylase complex, which inhibit the transcription of RP and RiBi genes [32,33,34]. Expression and biochemical analyses with mutants in these regulatory factors support their contribution to the translational response of yeast cells to iron depletion [12]. Moreover, the Sfp1 transcription factor activates the expression of RP and RiBi genes in normal conditions [35]. However, under iron scarcity, Sfp1 exits the nucleus, which is consistent with decreased RP and RiBi gene expression (Figure 1) [12]. Furthermore, the TORC1 inhibition caused by low iron reduces RNA Pol I and RNA Pol III activities, decreasing ribosomal RNA (rRNA) and tRNA expression because of lower expression of the RNA Pol I activator Rrn3 and dephosphorylation of the RNA Pol III repressor Maf1, respectively (Figure 1) [12]. All these processes converge to adapt global translation to iron bioavailability.

In eukaryotes, the rate-limiting step in translation is the initiation step [37]. Under optimal conditions, the first stage is the formation of the ternary complex composed of the translation initiation factor eIF2, GTP, and the initiator methionyl-tRNA (tRNA^Met^). After GTP hydrolysis, the ternary complex disassembles and the guanine nucleotide exchange factor eIF2B recycles eIF2-GDP into eIF2-GTP, and the subsequent addition of initiator tRNA^Met^ forms the ternary complex again [38]. Nevertheless, under nutrient starvation or other stress conditions, the presence of uncharged tRNAs activates yeast Gcn2 kinase via Gcn1–Gcn20 protein complex, facilitating the transfer of the uncharged tRNA from the ribosome to Gcn2 [39,40]. Active Gcn2 phosphorylates the alpha subunit of eIF2 (eIF2α), causing a drop in ternary complex formation and, consequently, an inhibition of global translation initiation. Recent cryo-electron microscopy data have described the interaction of Gcn1 protein with stalled and colliding 80S ribosomes [41]. This interaction could promote Gcn2 activation to repress global translation in response to environmental stresses. Bulk repression of protein synthesis is accompanied by enhanced translation of specific mRNAs encoding proteins that are required for the stress response. The best-characterized example is *GCN4* mRNA, which encodes for a transcriptional factor that activates the transcription of more than 500 genes, including genes involved in amino acid biosynthesis [42]. The global translational repression recently described for severe iron deficiency depends, at least partially, on the phosphorylation of eIF2α by Gcn2 and the Gcn1-Gcn20 complex (Figure 1) [36]. Unlike yeast, mammalian eIF2α subunit can be phosphorylated by four different kinases: PERK, PKR, GCN2, and heme-regulated eIF2α kinase HRI, which activate in response to different stimuli. HRI is predominantly expressed in erythroid cells and regulates translation in response to iron deficiency [43]. Recent data have shown the translational repression of mRNAs encoding cytosolic and mitochondrial RPs by HRI during iron deficiency, causing a decrease in cytosolic and mitochondrial protein synthesis [44]. Similar to yeast, eIF2α phosphorylation selectively enhances the translation of particular genes, including the transcription factor *ATF4*, which mitigates oxidative stress during erythroid differentiation [45]. Although additional and diverse mechanisms could be simultaneously regulating translation during adaptation to iron deficiency, regulation of the initiation step via eIF2 factor seems to be a common feature.

In *S. cerevisiae*, one of these additional mechanisms is mediated by the post-transcriptional factor Cth2, a protein highly expressed under iron deficiency and almost non-detectable in other growth conditions [10,11]. Cth2 is an RNA-binding protein, pertaining to the family of mammalian Tristetraprolin (TTP), which contains two highly conserved Cx_8_Cx_5_Cx_3_H tandem zinc fingers that specifically bind to adenosine/uridine (A/U)-rich elements (AREs) within the 3′ untranslated region (3′UTR) of many mRNAs that encode iron-containing proteins, promoting their decay and inhibiting their translation, seemingly at the initiation step [9,10,11,13]. Through its action, Cth2 coordinates a metabolic remodeling that allows yeast cells to prioritize the use of iron in essential processes, while other iron-dependent processes, such as respiration, are down-regulated [9,10,11]. A similar function for TTP in iron metabolism has been demonstrated in mammals [46,47]. On the other hand, under iron deprivation, other positive regulatory mechanisms could be in play to allow the translation of specific mRNAs. Thus, in addition to the activation of *GCN4* mRNA translation, which is observed under iron deficiency in an eIF2α-Gcn2-dependent manner, the translation of housekeeping genes, such as *ACT1*, is maintained up to long exposure to iron deficiency by an unknown mechanism [36].

## 3. The Relevance of Iron in Translation Elongation

### 3.1. Diphthamide Modification of the Translational Elongation Factor eEF2 Depends on Iron

The eukaryotic elongation factor 2 (eEF2), encoded by *EFT1* and *EFT2* in *S. cerevisiae*, is the functional and structural homolog of bacterial EF-G. Both are essential factors that catalyze the GTP-dependent translocation of the two tRNA molecules and the mRNA after peptide bond formation in the ribosome, when the newly formed peptidyl tRNA translocates from A to P site and the deacylated tRNA moves from the P to the E site [48,49]. In contrast to bacterial EF-G, both archaeal and eukaryotic EF2 factors undergo a unique and very conserved post-translational modification of a histidine residue, referred to as diphthamide (2-[3-carboxyamido-3-(trimethylammonio)-propyl] histidine). The name was assigned because this residue is the target for irreversible ADP ribosylation by diphtheria toxin, which renders eEF2 inactive, stopping protein synthesis and causing cell death (reviewed in [50]). A similar mechanism of action has been assigned to other ribosome-inhibiting protein toxins from plants, such as the antifungal agent sordarin, which has been used to identify yeast genes involved in diphthamide biosynthesis [51,52,53].

**Figure 2:**
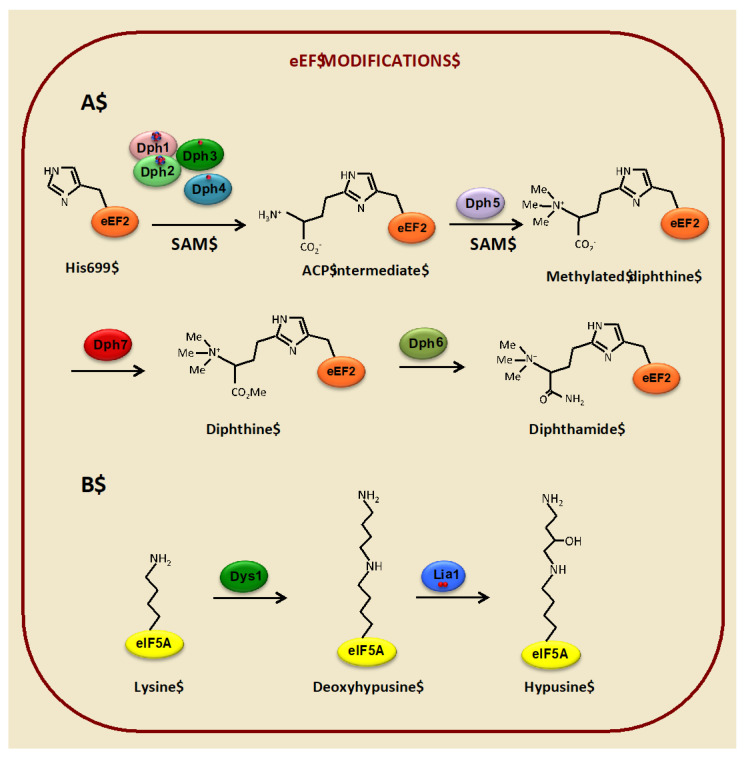
Iron-dependent proteins involved in the modification of the elongation factors eEF2 and eIF5A in the budding yeast *S. cerevisiae*. (**A**) Enzymatic steps in the diphthamide modification of eEF2. The ISCs in Dph1 and Dph2 are represented by red circles (iron nuclei) and blue circles (sulfur nuclei). Red circles in Dph3 and Dph4 represent mononuclear iron cofactors. Me means CH_3_. (**B**) Enzymatic steps in the hypusine modification of eIF5A. Red circles in Lia1 represent a binuclear iron cofactor.

In eukaryotes, diphthamide modification occurs through a four-step biosynthetic pathway, which comprises seven genes, denoted *DPH1-7*, whose functions have been mainly identified through studies in the yeast *S. cerevisiae* (reviewed in [15]) (Figure 2A). The first stage requires four iron-binding proteins (Dph1-4), which catalyze the transfer of a 3-amino-3-carboxypropyl (ACP) group from S-adenosyl-L-methionine (SAM) to the imidazole ring of a specific eEF2 histidine residue (H699 in yeast, H715 in mammals). The [4Fe-4S]-containing Dph1 and Dph2 paralogs form a heterodimer that directly transfers the ACP group from SAM to eEF2 through a reaction that involves non-canonical radical SAM chemistry [17,54,55]. Both clusters are required in vivo for diphthamide biosynthesis, but their functions are asymmetrical. Whereas the ISC in Dph1 is the catalytic site, the Dph2 ISC facilitates the reduction of Dph1 cluster by the Dph3-Cbr1-NADH system [56]. Dph3, also known as Kti11, co-purifies with Dph1-Dph2 and eEF2 and can bind both zinc and iron through a CSL zinc-finger domain [17]. In its iron-bound form, Dph3 serves as an electron donor to reduce and maintain active the ISCs of Dph1 and Dph2, while Cbr1 acts as a NADH-dependent Dph3 reductase [17,57]. Dph3 also acts as an electron carrier to many other proteins and processes, including Elp3 in the Elongator complex, thus being required for wobble uridine modifications of tRNAs (see below) [52,58,59]. The fourth partner, Dph4/Jjj3, also contains a CSL zinc-finger domain, as well as a DnaJ domain, and its human ortholog has been shown to bind iron, preferentially to zinc, and act as an electron carrier, which led assigning to this protein a similar role to Dph3 [18]. However, posterior studies in yeast failed to observe definite Dph4 iron-binding or electron carrier activity, so its exact function in diphthamide biosynthesis remains elusive [17]. Finally, three additional steps are required to complete diphthamide biosynthesis, each catalyzed by one enzyme. Dph5 is a methyl transferase, which uses SAM as the methyl donor, adding four methyl groups to the ACP-modified eEF2 to form methylated diphthine. Then, the methyl esterase Dph7 converts methylated diphtine to diphthine, and finally, the ATP-dependent enzyme Dph6 catalyzes the amidation step that converts diphthine to diphthamide [53,60,61].

While the pathophysiological role of diphthamide-eEF2 as the target for diphtheria and other toxins was established very early, the biological function of this modification is still under scrutiny. The elevated conservation from archaea to eukaryotes, of both diphthamide modification and its dedicated enzymatic pathway, highlights its importance for EF2 function, but eukaryotic cells lacking diphthamide-eEF2 are still viable. Thus, yeast *dph* mutants grow normally in most conditions, although substitution of histidine residue 699 causes growth defects under specific conditions [53,62,63,64]. On the other hand, negative genetic interactions have been described between EF2 (*EFT1*/*EFT2*) and *DPH1-7* gene deletions, with synthetic growth phenotypes caused by loss of diphthamide combined with eEF2 undersupply [65]. In mammalian cells, mutations that totally or partially abolish diphthamide modification lead to early developmental defects [66]. For instance, mutations in OVCA1 (the mammalian Dph2 ortholog, see Table 1) cause mouse embryonic lethality [67]. In humans, a diphthamide deficiency syndrome has been described, which causes developmental defects and ribosomopathy due to biallelic loss of function of *DPH2*, with a truncated protein that lacks a portion of the catalytic activity region, which includes a cysteine involved in [4Fe-4S] cluster binding [68]. Homozygotic mouse mutants for *DPH4* lack diphthamide modification and show abnormal development and perinatal death [69]. The more drastic effects of the absence of diphthamide observed in higher eukaryotes would be in accordance with the increasing evidence, showing that while yeast cells tolerate high levels of translational errors, through compensation by accelerated protein turnover, mammals are highly sensitive to even subtle disruptions of mRNA translation, which are in the basis of many developmental and neurological diseases, as well as tumorigenesis, supporting the notion that diphthamide modification plays an important biological role [70,71]. From the analysis of these mutants, it has become evident that diphthamide helps in maintaining translational fidelity by preventing -1 frameshifting during eEF2-mediated translocation, in both yeast and mammals [52,65,67,72].

Structurally, diphthamide is localized in the domain IV of eEF2, which acts as an anticodon mimicry domain, apparently contributing to proper codon–anticodon pairing and thus maintaining the accuracy of the translation process (reviewed in [15]). It has been proposed that lack of diphthamide could affect the translation of specific proteins, rather than global translation, which is maintained [69]. Effectively, recent studies with diphthamide-defective mammalian cells show reduced translation of selenocysteine-containing proteins, which are redox regulators that participate in the oxidative stress response. The mRNAs of this group of proteins harbor UGA codons that instead of being recognized as termination signals are decoded as selenocysteine. Interestingly, a model has been proposed where the location of diphthamide-eEF2 at the seleno-Cys/stop decision point would overlap with the binding site of the release factor ABCE1/Rli1 in a mutually exclusive manner, determining the fate of UGA-decoding codon, putatively in a redox-regulated manner through the redox reactive ISC of ABCE1 [73]. Additional studies support the role of diphthamide in maintaining the translation of specific proteins under stress conditions. Thus, in *S. cerevisiae*, *dph eft2* double mutants growth is compromised in response to thermal and chemical stresses, and a recent study in *Schyzosaccharomyces pombe* shows the involvement of eEF2 diphthamide modification, together with Elongator-mediated tRNA modifications, in maintenance of genome stability and response to different cytotoxic stresses [59,65]. In mammalian cells, IRES-dependent translation of specific proteins and adult stem cell proliferation depends on diphthamide under nutrient deprivation or chemical and oxidative stresses (reviewed in [74]). In addition to these stresses, and due to the conserved role of iron in the biosynthesis of diphthamide, it would be interesting to analyze how translational accuracy and/or translation of specific mRNAs is affected when yeast or mammalian cells are exposed to iron deficiency. These and other open questions regarding the importance of diphthamide-eEF2 in translation deserve future studies.

### 3.2. Hypusine Modification of the Translation Elongation Factor eIF5A Depends on Iron

The translation factor eIF5A is a small but indispensable protein highly conserved in eukaryotes. eIF5A is the only eukaryotic protein-containing hypusine (N-epsilon-(4-amino-2-hydroxybutyl)-lysine), a lysine- and polyamine-derived amino acid essential for its cellular function (reviewed in [75]). Most eukaryotes contain two similar but differentially expressed isoforms of eIF5A. In mammals, the major isoform, eIF5A1, is considered constitutive, being abundantly expressed in most cells and essential for cell proliferation, while eIF5A2 abundance is low in most normal tissues but highly expressed in many cancerous cell types [76]. In budding yeast, eIF5A is encoded by two paralog genes: *TIF51A* (also denoted as *HYP2*), which is the main isoform in aerobic conditions, and *TIF51B* (also known as *ANB1*), which is expressed under anaerobiosis. Depending on the genetic background, deletion of either *TIF51A* or both genes is lethal [77]. Both isoforms are functionally similar, as expression of either isoform under *GAL1* promoter allows growth in aerobic and anaerobic conditions [78,79]. Although eIF5A orthologs are present in both prokaryotes and archaea, their hypusination is only conserved in archaea.

eIF5A was initially described as a translation initiation factor, although its mild effect on global synthesis rate argued against this function [80]. Indeed, posterior studies with yeast eIF5A conditional mutants showed phenotypes more consistent with an involvement in translation elongation [81,82]. Specifically, eIF5A conditional mutants showed a polysome profile typical for translation elongation mutants, such as a decreased 80S peak and an increase in polysomes in the presence of cycloheximide, and a slower polysome run-off in the absence of cycloheximide. These observations led to redefining eIF5A as a translation elongation factor [81,82]. Posterior studies with the bacterial homolog of eIF5A, EF-P, supported a role of this factor in facilitating the formation of peptide bonds between consecutive proline residues, which in the absence of EF-P provoked the stalling of ribosomes [83,84,85]. Indeed, subsequent work in yeast supported a role for eukaryotic eIF5A in stimulating the translation of polyproline motifs [86,87]. Structural and hydroxyl radical mapping studies have shown that eIF5A adopts an L-shaped tRNA-like form and binds to the ribosome between the P and E sites, with the hypusine residue contacting residue Ala76 in the CCA-end of the P site tRNA, reorienting the nascent chain and facilitating the formation of the peptide bond [86,88,89,90]. In the absence of eIF5A, ribosomes would stall with the second or third proline codon situated in the P site [86].

More recent genome-wide studies of ribosome dynamics (5P-Seq and ribosome profiling) have shown that the absence of eIF5A provokes ribosome stalling not only at polyproline stretches, but also at many other tripeptide motifs, mainly containing proline, glycine, and charged amino acids, thus establishing that eIF5A acts as a general suppressor of ribosomal pausing at peptide sequences that are inefficiently translated [91,92]. These studies also suggested an involvement of eIF5A in facilitating translation termination. First, 5P-Seq data unveiled increased ribosome stalling at the stop codon in yeast thermosensitive mutants, which was independent of the presence of proline or other poorly translated amino acids [91]. Moreover, second, ribosome profiling in yeast cells depleted of eIF5A showed an increased stop codon peak that was not washed away with high salt, suggesting difficulties in the termination and release of the nascent peptide, although specific peptide sequences were not observed around the stop codon [92]. Further ribosome profiling experiments in human cells have shown that, in addition to its function as a ribosomal pause relief factor, eIF5A effects extend to translation initiation [93]. Specifically, eIF5A depletion increases translation within 5′UTR of a subset of stress response genes due to ribosome queuing after proximal ribosome pause sites, which would normally interfere with efficient scanning and start codon selection [93]. These data were supported by the analysis of previous yeast ribosome profiling data, as well as in human cells depleted of deoxyhypusine hydroxylase (DOHH), an enzyme involved in hypusination (see below) [92]. Altogether, these data extend our vision of eIF5A, uncovering a new role in maintaining the fidelity of translation initiation and a possible function in regulating translation under stress conditions. Even beyond translation, eIF5A has been described as a moonlighting protein, with other putative roles in RNA metabolism, such as nonsense-mediated mRNA decay or nucleo-cytoplasmic transport in archaea, yeast, and human cells [94,95,96,97]. If these tasks are derived or independent of their established role in translation is a question still open to debate.

Hypusination seems to be essential for all described eIF5A functions. The process of hypusination occurs in two well-conserved enzymatic steps, catalyzed by deoxyhypusine synthase (DHPS, encoded by *DYS1* in *S. cerevisiae*) and the aforementioned DOHH, encoded by *LIA1* in yeast, which is an iron- and oxygen-dependent enzyme (Figure 2B). Both enzymes are solely dedicated to the hypusination of eIF5A and are essential in most eukaryotes. In this sense, while DHPS is essential in archaea, a DOHH ortholog has not been found, with DHPS having both enzymatic activities in some cases [98,99]. As the deoxyhypusine formation by DHPS is reversible in vitro, it has been proposed that the hydroxylation step could have evolved as a stabilization mechanism for the deoxyhypusine modification [100,101]. Surprisingly, yeast *lia1∆* mutants are viable, which suggests that the deoxyhypusinated form of eIF5A is partially functional in yeast, as has been demonstrated for at least some eIF5A functions [102,103]. In fact, only deoxyhypusine-eIF5A, but not hypusine-eIF5A, is found in yeast *lia1∆* mutants, which indicates that the deoxyhypusine form can support growth [19,104]. Still, *lia1∆* mutants are unable to form shmoos in the presence of the alpha-mating factor, suggesting that the complete hypusination process is necessary for some eIF5A-dependent functions [87].

An intriguing question is why there are two isoforms of interchangeable eIF5A with different patterns of expression, both in yeast and mammals. One possibility not yet addressed is that each isoform could be affected differently by the degree of hypusination whose requirement would depend on environmental conditions. Yeast Lia1 and human DOHH are iron metalloenzymes containing a di-iron center that is essential for their activity, as well as for the maintenance of their structural integrity [19,20,21]. DOHH is also a conserved HEAT domain-containing protein, with eight HEAT repeats, distributed four in each arm (amino- and carboxy- terminal) of a symmetrical dyad. Four characteristic and strictly conserved His-Glu sites have been proposed to conform two Fe^2+^-binding sites that, through cooperation between the arms of the dyad, form a binuclear iron center at the active site. The hydroxylation of deoxyhypusine depends also on molecular oxygen, which is proposed to be coordinated by the two iron nuclei [20]. Loss of iron induces conformational changes in both human and yeast proteins and renders the enzyme inactive [20,21]. Despite iron being critical for Lia1 function, few studies have analyzed the effect of iron deficiency on *LIA1* expression and/or activity and how the hypusination levels influence eIF5A functionality. Early studies centered on the influence of oxygen and heme levels over eIF5A expression, showing a reciprocal regulation where *TIF51B* mRNA levels increase in anaerobiosis, whereas *TIF51A* expression is up-regulated during aerobiosis [78,105,106]. More recent studies have shown that *LIA1* mRNA levels also increase in anaerobiosis and that *TIF51A*, but not *TIF51B*, is required for growth under respiratory conditions, and its depletion reduces oxygen consumption [104,107]. Interestingly, full eIF5A hypusination is not required for maintaining high respiratory levels. *TIF51A* expression is regulated by TORC1 signaling under high glucose, and by Snf1 and the heme-dependent factor Hap1 under low glucose and respiratory conditions, respectively. Upon iron depletion, *TIF51A* and *LIA1* are down-regulated, whereas *TIF51B* is up-regulated, in a Hap1-dependent manner [12,104]. Several global expression studies in the pathogenic yeast *Candida glabrata* confirm the down-regulation of the corresponding Cg*LIA1* homolog under iron deficiency (reviewed in [108,109]). Interestingly, both *TIF51A* and *LIA1* mRNAs harbor potential AREs in their 3′UTRs. ARE-mediated decay of *TIF51A* has been involved in its regulation upon carbon source and, interestingly, Cth2 is an ARE-binding protein mRNA decay factor that functions under iron deficiency conditions (see above) [9,110]. Whether Cth2 regulates either *TIF51A* or *LIA1* under iron deprivation needs to be addressed in future studies.

Finally, the uniqueness of the hypusine modification of this fascinating translation factor makes it an attractive target to treat metastatic cancers (where eIF5A seems to be selectively up-regulated) or other conditions where the mitochondrial status of mammalian tissues is affected, such as ischaemic conditions or organ transplantations. Interestingly, the involvement of iron in its final hypusination step is opening the way to DOHH-targeted therapies, and the use of iron chelators such as ciclopirox or deferoxamine has already been shown to inhibit the growth of cancer cells ex vivo and in vivo, as well as to improve ischaemic conditions and the results of organ transplantation [111,112,113,114,115,116,117,118].

## 4. Iron Is Required for Translation Termination

### 4.1. The Conserved Iron-Containing Protein ABCE1/Rli1 Is Essential for Translation Termination and Ribosome Recycling and Reinitiation

ABCE1 was originally identified as an RNase L inhibitor [119]. However, its elevated conservation in all organisms except eubacteria rapidly questioned this function, since RNase L only occurs in mammals. ABCE1/Rli1 belongs to the ATP-binding cassette (ABC) subfamily of E proteins [120]. As typical ABC proteins, ABCE1/Rli1 harbor two head-to-tail oriented nucleotide-binding domains (NBDs) for ATP molecules within their carboxy-terminal region that are connected by a small and flexible hinge region. Their amino-terminal region displays two conserved and essential diamagnetic [4Fe-4S]^2+^ clusters with different electronic environments, one ferredoxin-like, and one unique ABCE1/Rli1-type cluster [121,122,123,124]. Initial studies in yeast demonstrated that the highly conserved cysteine residues coordinating both ISCs are important for cellular viability [6,121]. Most ABC members are implicated in ATP-driven unidirectional transport processes across biological membranes. Nevertheless, the predominantly cytoplasmic localization of ABCE1/Rli1 and the lack of any potential transmembrane domain pointed to an alternative function for these proteins [4,5,6,125,126,127]. Co-immunoprecipitation and co-sedimentation studies in yeast showing that Rli1 associates with ribosomes and translation initiation factors, including eIF2 and eIF3 complex components, eIF3j/Hcr1 subunit, and eIF5, as well as defects in the nuclear export of both ribosomal subunits and translational arrest upon Rli1 depletion, suggested that ABCE1/Rli1 proteins could be implicated in ribosome biogenesis and translation initiation [4,5,6]. Notably, the integrity of Rli1 ISC centers was essential for its functions in translation, demonstrating for the first time the molecular reason for the indispensable role that mitochondria play in cells, via ABCE1/Rli1 ISCs biosynthesis and assembly [5,6].

**Figure 3:**
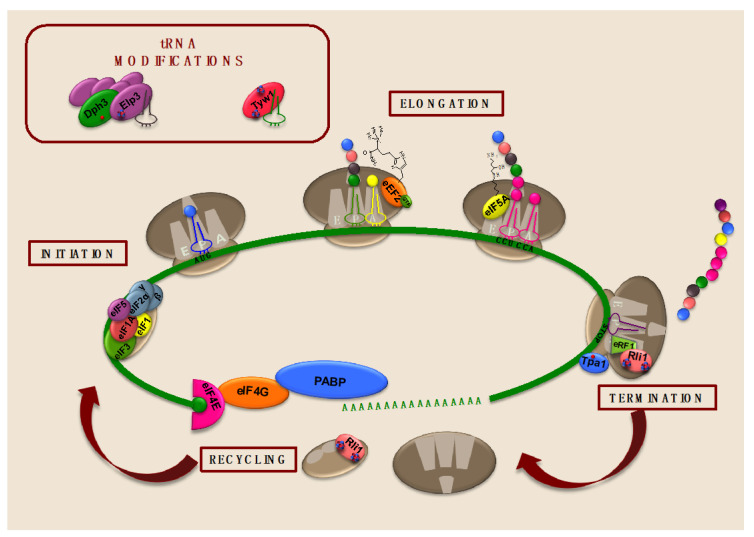
Iron-dependent proteins involved in the different steps of translation and in tRNA modifications in the budding yeast *S. cerevisiae*. In Rli1, Elp3, and Tyw1, ISCs are represented by red circles (iron nuclei) and blue circles (sulfur nuclei). Red circles in Dph3 and Tpa1 represent mononuclear iron cofactors.

Data from *Drosophila melanogaster* showing that a broad depletion of its ABCE1/Rli1 homolog did not alter 40S-bound eIF2 and eIF3 levels but reduced polysomes suggested that ABCE1/Rli1 could be also implicated in a step downstream of 43S complex assembly [128]. Subsequent studies with yeast, archaea, and human cells showing ABCE1/Rli1 interactions with either the eukaryotic or archaeal class I release factors e/aRF1, which structurally mimic a tRNA molecule, and the translational GTPase class II release factors e/aRF3 pointed to a central function in translation termination and ribosome recycling [122,129,130] (Figure 3). Structure and function analyses demonstrated a direct and stoichiometric interaction of an archaeal ABCE1 protein with the release factor aRF1, which stimulated an ISC- and ATP-dependent conformational change required to promote the splitting of ribosomes [122]. Similarly, in vitro reconstitution translation assays with eukaryotic components also unveiled that the dissociation and recycling of post-termination ribosomal complexes into free 60S subunits and tRNA- and mRNA-bound 40S subunits was promoted by ABCE1/Rli1 in a process that required ATP hydrolysis [130,131]. Biochemical, genetic, and ribosome profiling data also revealed that the class I release factor Dom34 (Pelota in humans) and the GTPase Hbs1—which have strong structural similarities to eRF1 and eRF3 release factors, respectively, and are implicated in the dissociation of empty or stalled 80S ribosome complexes on truncated mRNAs by eukaryotic quality-control surveillance mechanisms such as the no-go decay and non-stop decay—associate and are modulated by ABCE1/Rli1 in a mode slightly different from the canonical release factors, facilitating the dissociation and recycling of stalled ribosomes [123,131,132,133]. Unlike eRF1, Dom34 lacks the stop-codon recognition and the peptide hydrolysis motives. It has been proposed that Dom34-Hbs1 ribosome rescue is triggered by empty A sites [134]. Work with yeast confirmed that Rli1 physically and genetically interacts in vivo with translation termination factors eRF1 and eRF3, and, consistently, Rli1 down-regulation leads to defects in the recognition of stop codons and readthrough in an ISC-dependent manner [129] (Figure 3). Consistent with these data, ribosome profiling experiments with yeast Rli1-defective cells showed the accumulation of 80S ribosomes at the stop codon and its adjacent 3′UTR, which aberrantly reinitiated translation of small peptides without the need of a canonical start codon [135]. Yeast ribosome profiling assays also demonstrated the role that Dom34 plays in recycling ribosomes that escape stop codon and enter downstream non-coding regions [135,136]. In this sense, defects in ISC delivery to ABCE1 due to oxidative stress or lysosomal dysfunction cause stop codon readthrough and ribosome movement into 3′UTRs [137]. Thus, both ABCE1/Rli1 and Dom34/Pelota are necessary for the access of post-transcriptional regulatory RNA-binding proteins to the 3′UTR of mRNAs since the movement of a single ribosome through the 3′UTR in ABCE1-defective cells is sufficient to displace mRNA-bound proteins [137]. Recent data have demonstrated that non-sense mediated decay and post-transcriptional regulatory pathways such as microRNA and ARE-mediated decay are impaired upon depletion of ABCE1 [137].

Both translation reconstitution assays and structural data on eukaryotic and archaeal translation termination complexes at different stages, as well as biochemical and genetic analyses with yeast, mammalian, and archaeal orthologs, have tremendously contributed to our current model for ABCE1/Rli1 function in the dissociation of ribosomal complexes during translation termination and the reuse of their components (Figure 3) [122,123,131,138,139]. According to these data, eukaryotic translation termination can be divided into the following stages [138,140,141,142]. First, eRF1-eRF3 or Dom34-Hbs1 heterodimers recognize and bind to the A ribosomal site of pre-termination complexes or stalled ribosomes, respectively. Then, GTP hydrolysis by eRF3/Hbs1 promotes their dissociation and a conformational change that allows ABCE1/Rli1 association to the GTPase-binding site in a semi-closed conformation, establishing contacts with both ribosomal subunits. In the next step, ABCE1/Rli1 accommodates eRF1 close to the CCA-end of the P-site tRNA to facilitate peptide release during canonical termination, or positions Dom34 in the proximity of the peptidyl transferase center during quality control surveillance processes [123]. Finally, ATP-binding triggers a conformational change of ABCE1/Rli1 from an open state to a closure or dimerization of both NBDs that occludes ATP and causes an extensive rotation of the ISC domain towards a groove between the S12 protein and the rRNA of the small ribosomal subunit, causing a collision with A-site factors that tears ribosomal subunits apart [122,138,139]. The ISC domain remains bound to the small ribosomal subunit until ATP is hydrolyzed, which causes an opening of the NBDs and allows the ISC domain to swing back to its original position [138]. In contradiction with this model, other studies have proposed that ATP hydrolysis is also necessary to split ribosomal complexes [123,130,131]. It seems that various rounds of ATP binding and hydrolysis could be necessary until the final post-splitting state is achieved [130,141].

ABCE1 anchoring to the translational GTPase-binding site after ribosome splitting, as a component of the 43S initiation complex, has been proposed to prevent ribosomal subunit association before the proper assembly of initiation factors [138,139]. Low-resolution cryo-electron microscopy reconstitution data even suggest that ABCE1 adopts a novel conformation to be a component of late-stage 48S translation initiation complexes, acting as a bona fide initiation factor with potential functions in stabilizing the binding of other initiation factors, limiting the association of ribosomal subunits and acting as an energy-sensing translational regulator [14,143]. In vitro reconstitution data with human components and in vivo yeast assays have also implicated translation initiation factors such as eIF3 and eIF3j/Hcr1 in the dissociation of post-termination complexes, leading to stop codon readthrough when defective, thus coupling translation termination, recycling, and reinitiation of further rounds of translation via ABCE1/Rli1 [135,144,145]. Although structural studies are necessary to decipher its precise task in ribosome biogenesis, yeast Rli1 has been also implicated in a quality control system during the maturation of small ribosome subunits [146]. The translation initiation GTPase eIF5B facilitates the association of mature large ribosomal subunits to pre-40S subunits. However, since the assembled ribosomes are not competent for translation initiation because they lack mRNA or initiator tRNA, Rli1, and Dom34 break apart the 80S-like subunits into their components. It has been proposed that this translation-like cycle that precedes the canonical translation initiation process prepares maturing 40S ribosomes for translation initiation since it tests critical activities of the maturing 40S subunit [146]. Altogether, these data strongly indicate that ABCE1/Rli1 is a multi-task factor that coordinates the different phases of protein synthesis.

According to multiple experimental data, ISC domains play a fundamental structural function in the ABCE1/Rli1-mediated dissociation of ribosomes and its subsequent anchoring to the small ribosomal subunit. The organization and redox potentials of both ISCs do not support a redox-catalytic role for these clusters [121]. However, ISCs destined for insertion into yeast Rli1 protein seem to be the primary in vivo target for reactive oxygen species (ROS) prior to their assembly, whereas they are not solvent exposed but protected when incorporated into the holoprotein [136,147]. Increasing the expression of *RLI1* ameliorates ROS resistance, whereas its down-regulation has the opposite effect, suggesting that Rli1 ISCs do not serve as oxidative stress sensors, with these phenotypes probably being just the deleterious consequence of oxidative damage [147]. Although iron is essential for most ABCE1/Rli1 tasks, little is known about potential mechanisms that regulate or compensate their functions in response to iron-defective conditions. Interestingly, homologs of *DOM34* and *HBS1* in the pathogen yeast *Candida glabrata* are required for optimal growth in iron-limited environments [148]. In fact, the expression of *C. glabrata DOM34* and *HBS1* is up-regulated in response to iron depletion via the iron-responsive transcription factor Aft1 [148]. It is tempting to speculate that *C. glabrata* may increase the expression of Dom35 and Hbs1 to compensate for potential translation termination defects caused by a decrease in the activity of ABCE1/Rli1 during iron deficiency. ABCE1 function is also lost during the maturation of red blood cells, probably because its ISC cannot be synthesized after mitochondrial breakdown, causing the accumulation of post-termination non-recycled ribosomes on 3′UTRs of mRNAs [149]. Thus, induction of the ribosome recycling factors Pelota and Hbs1 is required to compensate ABCE1 loss of function and support protein synthesis during the terminal differentiation of red blood cells [149]. In *S. cerevisiae*, Dom34 rescue of ribosomes in 3′UTRs is exacerbated in Rli1-defective cells, although no induction of *DOM34* and *HBS1* has been observed [135]. In this sense, further studies are necessary to test whether cells possess specific mechanisms to optimize or replace ABCE1/Rli1 function in response to cofactor-delivery defects or iron limitation.

### 4.2. The Iron-Dependent Prolyl Hydroxylase Tpa1 Modulates Translation Termination

Fe^2+^/2-oxoglutarate (2OG) dioxygenases constitute a largely conserved family of proteins that catalyze a wide diversity of enzymatic reactions and participate in multiple biological functions [150]. In yeast, Fe^2+^ and 2OG dioxygenase Tpa1 catalyzes two sequential hydroxylations of a highly conserved proline residue in the Rps23 subunit of the 40S ribosomal decoding center to control translation termination efficiency and accuracy [24,25]. Substrate hydroxylation requires the oxidation of Fe^2+^ to Fe^3+^/Fe^4+^-superoxo species and the coupled decarboxylation of 2OG co-substrate to succinate and CO_2_. Ascorbate and glutathione reduce iron back to its ferrous state [151]. The translational control exerted through Rps23 oxidation seems to be conserved in eukaryotes including humans and fission yeast, although in these cases it results in Rps23 mono-hydroxylation [26,152].

Various studies have contributed to solving the structure of the *S. cerevisiae* Tpa1 and its human homolog OGFOD1 [153,154,155]. Yeast Tpa1 is composed of two domains of unequal size with a characteristic double-stranded β–helix fold known as jelly-roll fold [153,154]. The small amino-terminal Fe^2+^ and 2OG-dependent dioxygenase domain is similar to prolyl hydroxylases such as human OGFOD1 and binds a mononuclear non-heme Fe^2+^ via three protein residues comprising a conserved Hx(D/E)…H motif, whereas the large carboxy-terminal domain exhibits a similar overall fold but does not bind iron and 2OG [153,154]. Structural and functional studies indicate that Tpa1 can adopt a reversible cylinder-like homodimeric conformation in solution [153,154].

Yeast Tpa1 protein interacts in vivo with the translation release factors eRF1 and eRF3, and poly(A)-binding protein Pab1 in an RNA-independent manner (Figure 3) [24]. Thus, yeast cells defective in Tpa1 catalytic activity or in its carboxy-terminal interacting partner protein Ett1 display increased stop codon readthrough [24,154]. Importantly, both molecular oxygen and its Fe^2+^-cofactor are essential for Rps23 hydroxylation and translation termination accuracy [25]. It is tempting to speculate that, as described for Rli1, ribosomal readthrough could cause the removal of RNA-binding proteins from 3′UTRs. Consistent with this, yeast *tpa1∆* cells exhibit altered mRNA half-lives and poly(A) tail lengths [24]. Stop codon readthrough assays in yeast suggest that Tpa1 regulates translation termination accuracy in a sequence-context-dependent manner, raising the question of whether Tpa1 regulates specific sets of genes [25]. The elucidation of the molecular mechanism and specific function of Tpa1 and other prolyl hydroxylases in translation termination requires further work.

## 5. Iron-Dependent Enzymes Catalyze tRNA Modifications Important for Translation

Post-transcriptional modifications of tRNAs are required for proper translation and can act as regulatory mechanisms of translational control in response to cellular stresses due to dynamic changes in tRNA levels. Recently, numerous studies have established that deregulation of tRNA modifications could be associated with cancer and other human mitochondrial disorders [156,157]. Interestingly, particular tRNA modifications rely on iron-dependent proteins (Figure 3).

### 5.1. The Fe/S Cluster Enzyme Tyw1 Is Important for Wybutosine Modification of tRNA^Phe^ and Protection against High-Iron Conditions

Wybutosine modification on the 3′-position adjacent to the anticodon of the phenylalanine tRNA (tRNA^Phe^) in eukaryotes and archaea was one of the earliest tRNA modifications to be discovered. This modification plays a crucial role in the codon–anticodon stabilization to maintain the reading frame and prevent translational misreading. Wybutosine is a fluorescent tricyclic base derived from guanosine. In yeast, the initial step in its biosynthetic pathway consists of the modification of guanosine to N^1^-methylguanosine (m^1^G^37^-tRNA) by Trm5 methylase. Then, the radical SAM enzyme Tyw1 promotes the synthesis of the imidazoline ring containing 4-demethylguanosine (ImG^14^-tRNA). Subsequent steps catalyzed by the Tyw2-Tyw3-Tyw4 enzymatic complex finally lead to wybutosine [22,158,159].

Structural studies in archaea have shown that Tyw1 protein is composed of a radical SAM domain with an (α/β)_6_ core structure containing two [4Fe-4S] clusters coordinated by conserved Cx_3_Cx_2_C motifs and the catalytic pocket in the center of a partial barrel architecture [160,161] (Figure 3). Eukaryotic Tyw1 proteins possess an additional amino-terminal flavodoxin-binding domain involved in electron transport and have been localized at the cytosolic surface of the endoplasmic reticulum [23]. One of Tyw1 ISCs acts as a strong reducing agent to cleave SAM into methionine and highly reactive 5′dA radical species, which triggers the H-atom subtraction from the methyl group of m^1^G^37^-tRNA. The second ISC functions as an oxidative agent and interacts with pyruvate [162]. According to recent in vitro ^13^C pyruvate isotopologue assays, pyruvate could act as the donor of two carbons incorporated into the imidazole ring of the tRNA [163]. Yeast in vivo assays have confirmed the fundamental role of both iron centers in the formation of wybutosine since defects in iron cofactor delivery or mutagenesis of the conserved cysteines involved in iron coordination abolish the catalytic activity of Tyw1 and the synthesis of wybutosine [22,160].

Various observations also suggest that Tyw1 could protect yeast cells from high iron toxicity by sequestering cytosolic-free iron [23]. First, the expression of *TYW1* gene is transcriptionally activated in response to high-iron conditions via the iron-protective Yap5 transcription factor. Second, deletion of *TYW1* increases the sensitivity to iron of yeast cells lacking the vacuolar iron detoxification importer Ccc1. Third, overexpression of *TYW1* activates the iron regulon, including the cell surface iron acquisition system, leading to an increase in intracellular iron levels and a substantial growth defect. The mechanism for *TYW1*-mediated activation of the iron regulon is currently unknown but depends on a Tyw1 amino-terminal region containing its endoplasmic reticulum anchoring domain and a *Saccharomycetaceae*-specific motif [23]. Importantly, deletion of *TYW2*, which encodes a tRNA methyltransferase, whose substrate is the product of Tyw1, does not lead to any iron-related phenotype, suggesting that Tyw1 function in wybutosine biosynthesis is not responsible for its role in high iron protection [23]. Given that the overexpression of either the ISC-containing SAM domain of Tyw1 or another ISC-containing protein, such as Leu2, protects *ccc1∆tyw1∆* cells from cytotoxicity, it has been proposed that sequestration of ISCs into Tyw1 could protect cells from high iron toxicity [23]. Thus, Tyw1 could represent a dual-function protein implicated in wybutosine synthesis and high-iron protection.

### 5.2. The Elongator Fe/S Cluster Subunit Elp3 Enhances Translation Efficiency by Catalyzing tRNA Wobble Uridine Modifications

Elp3 is an ISC-containing catalytic subunit of the eukaryotic Elongator complex, which was initially implicated in transcription elongation because of its association with phosphorylated RNA Pol II and its Elp3-dependent histone/lysine acetyltransferase activity [164,165,166,167,168]. However, further studies strongly indicate that the physiologically relevant function of Elongator is a wobble uridine modification of particular tRNAs [169]. Elongator consists of a multisubunit complex composed of two catalytic Elp1-Elp2-Elp3 (Elp123) modules and two Elp4-Elp5-Elp6 (Elp456) subcomplexes [169,170,171]. Electron microscopy and crosslinking mass spectrometry with all six different *S. cerevisiae* Elongator subunits have revealed an asymmetric overall holoElongator structure in which the Elp456 hexamer assembles to one side of the two Elp123 modules, so that both Elp3 subunits are located in different environments [172,173]. Despite the mechanism for this asymmetrical assembly not being known, the complete integrity of the complex has been demonstrated to be important for function [169].

Bacterial and archaeal Elp3 proteins, which are conserved in all the kingdoms of life, contain an amino-terminal radical SAM domain with a [4Fe-4S] cluster and a carboxy-terminal lysine acetyltransferase (KAT) domain that share a large interface harboring a tRNA-binding pocket and the active site of the enzyme [167,168,174] (Figure 3). Thus, Elp3 functions as a non-canonical tRNA acetyltransferase that catalyzes the initial post-transcriptional 5-carbamoylmethyl (cm^5^) modification of the wobble position U_34_ within the anticodon stem loop-decoding region of tRNAs recognizing AA-ending codons, which stabilizes codon–anticodon association and facilitates translation [58,174,175]. In vivo and in vitro assays with yeast mutants lacking conserved cysteine residues that coordinate Elp3 ISC have demonstrated that the iron cofactor is essential for the structural integrity of Elongator and consequently for its catalytic activity [168,176]. Although the mechanism for tRNA modification and substrate specificity has not been fully elucidated, the available structural and biochemical data suggest the following general model: (1) a tRNA molecule initially binds to the Elp456 ring, whose ATPase activity promotes its dissociation and transfer to an Elp123 subcomplex; (2) tRNA-binding to Elp3 promotes a structural rearrangement that facilitates acetyl-CoA association, hydrolysis, and transfer to Elp3 KAT domain; (3) Elp3 recruits SAM, which is converted into methionine and a 5′deoxyadenosine (5′dA) radical, potentially via its ISC; (4) then, 5′dA interacts with the acetyl group and generates an acetyl radical that is transferred to the C5 position of the U_34_ tRNA to obtain the modified tRNA that leaves the complex.

Studies in both budding and fission yeasts have implicated Elp3 in the response to multiple cellular stresses, probably due to Elp3-deficiency-associated defects in the translation of specific regulatory factors with lysine codons biased towards the AAA codon, whereas highly expressed genes seem to bypass Elp3 requirement by enhancing the AAG to AAA lysine codon ratio. In *S. cerevisiae*, defects in Elongator including *ELP3* deletion impair the translation of mitochondrial proteins at 37 °C, decreasing their cellular respiratory capacity. Multiple rescue assays have demonstrated that this phenotype is due to defects in tRNA^Lys^_UUU_ modifications [177]. In *S. pombe*, Elp3 is important for oxidative stress survival because it facilitates the translation of stress transcription factors, including Atf1 and Pcr1, which are enriched in lysine codons AAA as compared to AAG [178]. Moreover, budding yeast *elp3∆* mutants are also sensitive to methyl methansulfonate and display growth defects at low temperatures [59]. In all cases, overexpression of tRNA^Lys^_UUU_ suppresses the Elp3-associated phenotypes, indicating that its defects are a consequence of decreased tRNA^Lys^_UUU_ uridine modifications [59,177,178]. A proteome-wide study in *S. pombe* has shown that Elp3 is required for the efficient translation of genes with a high content in the lysine AAA codons, which includes genes implicated in cell cycle control due to a defect in the translation of Cdr2 kinase, a central regulator of mitosis [179]. This relatively general role of Elongator in maintaining proper cellular protein homeostasis could explain the pleiotropic phenotypes associated with Elp3 defects. In humans, defects in Elongator function that alter its tRNA modification activity have been associated with cancer and neurodegenerative disorders [168,180,181].

## 6. Conclusions and Future Perspectives

Iron is a redox-active element that participates as a protein cofactor in many metabolic pathways. Fundamental findings, mostly from the last decade, have positioned iron at the center of protein synthesis. As described above, iron-containing proteins are implicated in most steps of the translation process, including ribosome biogenesis and recycling, tRNA modification, and the stages of initiation, elongation, and termination. Although required for function, the specific role played by this metal cofactor has not been deciphered in most cases. Further structural and biochemical studies are necessary to position iron as either a modulator of protein conformation and structure or an electron transfer factor.

Many studies in both human and yeast cells have shown that protein synthesis diminishes in response to environmental stresses and nutritional deficiencies, including iron starvation. However, little is known about the mechanisms that eukaryotic cells utilize to modulate translation when iron bioavailability decreases. Studies in yeast have demonstrated that other essential iron-dependent processes, such as the de novo synthesis of deoxyribonucleotides by ribonucleotide reductase, possess multiple overlapping mechanisms to prioritize and maintain enzymatic activity when iron cofactor availability decreases (reviewed in [8]). Whether specific regulatory mechanisms optimize the function of iron-dependent proteins implicated in translation during iron deficiency is widely unknown. Using yeast to study iron-cofactor delivery and other regulatory strategies could tremendously contribute to elucidate specific methods that eukaryotic cells employ to modulate protein synthesis during the progress of iron starvation.

## Figures and Tables

**Table 1:** List of the iron-containing enzymes involved in translation in the yeast *S. cerevisiae*. Human homologs, functions, and references are indicated.

Yeast	Human	Function	References
Rli1	ABCE1	Ribosome biogenesis and recycling/ Translation initiation and termination	[6,14]
Dph1	DPH1	Diphthamide biosynthesis	[15]
Dph2	DPH2L1/OVCA1	Diphthamide biosynthesis	[15,16]
Dph3/Kti11	DPH3	Diphthamide biosynthesis/ Subunit of Elongator complex	[15,17]
Dph4	DPH4	Diphthamide biosynthesis	[15,17,18]
Lia1	DOHH	Deoxyhypusine hydroxylase	[19,20,21]
Tyw1	TYW1A/RSAFD1	Synthesis of wybutosine in tRNA^Phe^/Translation accuracy/Protection against high iron conditions	[22,23]
Tpa1	OGFOD1	Prolyl hydroxylation of Rps23/Translation termination/DNA alkylation repair	[24,25,26,27]
